# Transnasal endoscopic closure of persistent gastrocutaneous fistula via percutaneous suturing in altered oropharyngeal anatomy

**DOI:** 10.1055/a-2462-2023

**Published:** 2024-11-13

**Authors:** Agnieszka Maniak, Kanika Garg, Gaurav Kakked, Ajaypal Singh, Irving Waxman, Christopher G. Chapman, Neal A. Mehta

**Affiliations:** 1 2468 Center for Interventional and Therapeutic Endoscopy, Division of Digestive Diseases and Nutrition, Rush University Medical Center, Chicago, United States

A 65-year-old man with a past medical history of oropharyngeal squamous cell carcinoma, status post resection and flap reconstruction, presented with concern for gastrojejunostomy tube malfunction. During the patient’s most recent gastrojejunostomy exchange, he was noted to have significant leakage around the tube. On physical examination, there was a 15-mm gastrocutaneous fistula visualized on the abdominal wall with an ulceration lateral to the tract. The gastrojejunostomy was removed at bedside in the hope of healing the ulceration and fistula.

The aim of this procedure was to use percutaneous suturing under direct endoscopic visualization to close a large gastrocutaneous fistula. Owing to distorted oropharyngeal anatomy, a pediatric endoscope was introduced through the left naris ( Video 1 ). A large 15-mm gastrocutaneous fistula was noted in the gastric body. Brush and biopsy forceps were used to disrupt the tract to create granulation tissue. To close the defect, two 16-gauge angiocath needles were passed percutaneously through the abdominal wall into the stomach under direct endoscopic visualization on either side of the gastrocutaneous fistula. The needles were removed and a 2–0 monocryl suture was passed through one of the catheters from the outside. A micro biopsy forceps was passed through the other catheter and grasped the suture in the stomach under direct endoscopic visualization. The suture was then pulled through the abdominal wall with the forceps. Multiple surgical knots were tied on the cutaneous side to close the defect.

Transnasal endoscopic closure of persistent gastrocutaneous fistula via percutaneous suturing in altered oropharyngeal anatomy.Video 1

A similar approach to a persistent gastrocutaneous fistula was presented by Garcia et al. 1 . The suture was reinforced with an endoscopic suturing device, which was not feasible in our case given the transnasal approach limiting endoscopic therapies. This case portrays a successful technique for closure of a gastrocutaneous fistula in a patient with altered oropharyngeal anatomy.

Endoscopy_UCTN_Code_TTT_1AO_2AK

## Notes

Endoscopy E-Videos https://eref.thieme.de/e-videos
*E-Videos* is an open access online section of the journal *Endoscopy* , reporting on interesting cases and new techniques in gastroenterological endoscopy. All papers include a high-quality video and are published with a Creative Commons CC-BY license. Endoscopy E-Videos qualify for HINARI discounts and waivers and eligibility is automatically checked during the submission process. We grant 100% waivers to articles whose corresponding authors are based in Group A countries and 50% waivers to those who are based in Group B countries as classified by Research4Life (see: https://www.research4life.org/access/eligibility/ ). This section has its own submission website at https://mc.manuscriptcentral.com/e-videos .
